# Performant barcode decoding for herbarium specimen images using vector‐assisted region proposals (VARP)

**DOI:** 10.1002/aps3.11436

**Published:** 2021-06-03

**Authors:** Caleb Powell, Joey Shaw

**Affiliations:** ^1^ Department of Biology, Geology and Environmental Science University of Tennessee at Chattanooga 615 McCallie Avenue Chattanooga Tennessee 37403 USA

**Keywords:** barcode, biodiversity data, digitization, herbarium, natural history collections

## Abstract

**Premise:**

The scale and associated costs of herbarium digitization make process automation appealing. One such process for many workflows is the association of specimen image files with barcode values stored with the specimen. Here, an innovation is presented that improves the speed and accuracy of decoding barcodes from specimen images.

**Methods and Results:**

Geometric features common in barcodes are used to identify the regions of specimen images that are likely to contain a barcode. The proposed regions are then combined into a significantly reduced composite image that is decoded using traditional barcode reading libraries. Tested against existing solutions, this method demonstrated the highest success rate (96.5%) and the second fastest processing time (617 ms).

**Conclusions:**

This method was developed to support a larger effort to automate specimen image post‐processing in real‐time, highlighting the importance of execution time. Although initially designed for herbarium digitization, this method may be useful for other high‐resolution applications.

The digitization of natural history collections continues to generate a large quantity of specimen images. As of May 2021, the iDigbio portal (https://www.idigbio.org) is serving over 26 million preserved plant specimen records with associated media, most of which are images. It is estimated that the available records represent a relatively small proportion of the data that remain to be mobilized (i.e., making digital representations of specimens available in online portals) (Vollmar et al., 2010; Barkworth and Murrell, 2012; Ariño, 2018). The scale of natural history digitization is motivating innovations in process automation both holistically (Sweeney et al., 2018) and on a process‐by‐process basis (Hudson et al., 2015; Powell et al., 2019; Ledesma et al., 2020). Here, one such innovation is presented that was developed to improve the speed and accuracy with which barcode values may be decoded from within herbarium specimen images.

After being captured, herbarium specimen images are paired with a record containing additional information related to the specimen (e.g., transcribed label data, phenological state, specimen provenance). Common workflows for matching images to their records suggest naming image files according to a catalog number that is encoded on a barcode and stored with the specimen (Nelson et al., 2015). A few methods exist that facilitate naming specimen images accordingly. Sequential naming schemes are the most direct, using camera preferences to generate file names that correspond to the catalog numbers of the specimens being imaged. This method assumes images are captured in the order that sequential barcodes were applied and is therefore highly vulnerable to synchronization errors, such as when one specimen is accidentally skipped, thus causing all subsequent images to be named incorrectly. Another method used to name specimen image files integrates a handheld barcode scanner and custom user dialog box into the imaging process (Consortium of Pacific Northwest Herbaria, 2021). This unfortunately imposes an additional step for the operator, slowing the imaging process somewhat. Due to the drawbacks associated with these methods, automated solutions have been explored that assign a file name by decoding barcodes from within specimen images.

Multiple solutions exist for decoding barcodes from images, and previous works have demonstrated their usefulness in natural history digitization. Sweeney et al. (2018) used the open‐source Java library “ZXing” (https://github.com/zxing/zxing) to automate barcode decoding in a high‐throughput herbarium digitization system, and the natural history image processing software suite *Inselect* (Hudson et al., 2015) uses the open‐source C library “Zbar” for barcode decoding. *Inselect* was written in Python and accesses Zbar using a compatibility library (i.e., a "wrapper”) called PyZbar (Hudson, 2016), which exposes a limited set of Zbar functions to the Python environment. Both *Inselect* and PyZbar are now available through the Natural History Museum of London’s GitHub account (https://github.com/NaturalHistoryMuseum). Each of these methods detect barcode patterns by “line scanning,” which evaluates sequential pixels along an image’s rows or columns to determine if the pattern of light and dark pixels corresponds to a barcode value. In the case of Zbar, images are evaluated both row‐wise and column‐wise to accommodate for unknown orientations (i.e., portrait or landscape). This accommodation comes at the cost of evaluating each pixel twice, once in the horizontal context and again in the vertical. Because line scanning is limited to these horizontal and vertical contexts, it is rotationally variant, meaning these methods fail when the entire barcode pattern does not lie along a single row or column. One workaround to this limitation is to repeatedly line scan while performing a series of rotations on the image until a barcode value is detected. This workaround has a number of drawbacks: (1) the transformations and additional line scanning incur additional computation time, (2) it may fail to detect multiple barcodes within an image if they are present at differing angles, and (3) this method delays identification when no barcode is detectable.

The limitations of traditional line scanning are typically negligible in other, more typical use cases where the barcode is the focal point of a relatively low‐resolution image. Herbarium images, on the other hand, are usually high resolution—often exceeding 24 million pixels—which increases the computation costs associated with line scanning. Furthermore, because barcodes on herbarium specimens only serve to facilitate curation, their size relative to the specimen has understandably been kept minimal. This makes traditional resolution reduction strategies infeasible as they discard much of the barcode pattern. Inspired by region proposal networks (RPNs), a novel protocol is presented that improves upon traditional line scanning by making it more computationally efficient and rotationally invariant. RPNs leverage faster, less precise methods to select specific regions of an image that are suitable for more intensive evaluation. The vector‐assisted region proposal (VARP) method presented here uses computer vision techniques to identify arbitrarily oriented lines (i.e., vectors) in an image that may contain barcode data, then organizes and evaluates only those regions through line scanning. This efficient, rotationally invariant method is demonstrated to be more performant at decoding barcodes than available alternatives.

## METHODS AND RESULTS

### Vector identification

**FIGURE 1:**
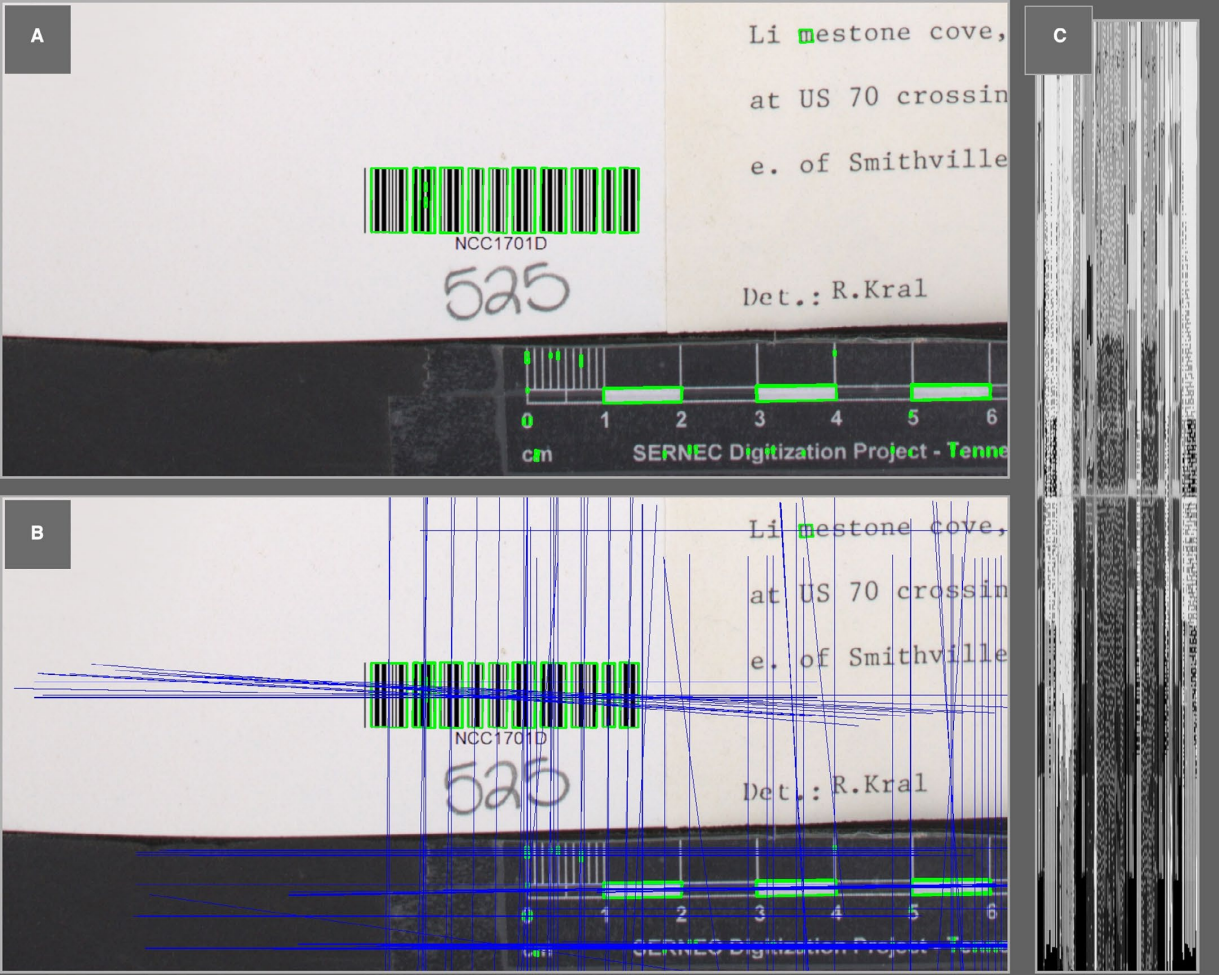
A visualization of the VARP method for identifying barcodes. (A) Green boxes illustrate the rectangles identified by VARP’s contour detection. (B) Blue lines illustrate vectors identified for pixel sampling. (C) The resulting composite produced by vertically concatenating the pixels sampled along each vector.

VARP identifies candidate regions by exploiting geometric tendencies in the single‐dimensional barcodes (e.g., traditional Code 39 and Code 128 formats that appear as parallel black and white lines) common in natural history collections. Fundamentally, these barcodes are composed of a series of rectangles with varying widths. OpenCV (https://opencv.org) is used to convert images to grayscale and detect rectangle‐shaped contours (Fig. 1A) within them. Contour detection is not influenced by geometric rotation and is therefore not rotationally variant. Due to the relatively low consequences of incidental false positives, the parameters used to differentiate rectangles from other contours are permissive enough to handle moderate distortions associated with imperfect imaging conditions. For each rectangle identified, VARP calculates a vector that intersects the midpoints of the parallel edges. To ensure the vectors intersect the entire barcode pattern, they are then extended in both directions for a length relative to one‐sixth of the image’s smallest dimension (Fig. 1B). Calculating extension lengths relative to the image size makes the process robust to varying resolutions. The specific extension parameters (i.e., one‐sixth the smaller dimension) were chosen based on typical vascular plant herbarium specimen image parameters, but this is a user‐defined parameter set by “‐‐extension_value”, which can be customized to specific specimen types. These default parameters assume a relative maximum width that a barcode might occupy within an image and should be reevaluated for applications with larger relative barcode sizes such as bryophyte packets and fungi label images. Because the entirety of each vector is being proposed for further analysis, excessively large extension parameters would incur unnecessary computation costs during that analysis.

### Composite image generation

Cartesian coordinates are calculated to approximate the location of all pixels intersected by each vector. The values (i.e., relative grayness) of those pixels are then vertically concatenated into a matrix forming a new composite image (Fig. 1C). Organizing this composite image has three advantages: (1) otherwise differentially angled lines are aligned along a shared axis, which overcomes rotational variance; (2) this shared orientation makes row‐wise line scanning unnecessary; and (3) the composite image is greatly reduced in size. Composite images have a pixel width equal to the number of proposed vectors and a height equal to the longest vector, which is approximately two times the vector extension value. For example, if 200 rectangles are identified in a 4000 pixel by 6000 pixel image and the default extension values are used, then the resulting composite would be 200 pixels by 1333 pixels, reducing the total pixels by 98.9%. This diminished resolution retains the most meaningful dimension of any correctly identified barcode patterns while drastically reducing the total pixels evaluated during the final line scanning step.

### Line scanning

Barcodes are then decoded from the composite image using line scanning. To leverage this vertical orientation, the PyZbar library was modified to expose single‐axis decoding parameters, which are used to deactivate row‐wise line scanning (https://github.com/CapPow/pyzbar). If no barcodes are initially decoded, multiple successive attempts are made while increasing the composite’s contrast and brightness. When performed on the much smaller composite image, these additional manipulations are computationally inexpensive, yet these fallback procedures do assume that the barcode is present in the composite.

### Method evaluation

VARP’s performance was compared to alternatives using a test data set of 1000 herbarium records. Data gathering and evaluations were performed using Python 3.8 (https://www.python.org) in a Jupyter notebook (https://jupyter.org). All evaluations were performed on a 3.6 GHz Intel Core i7‐3520M processor (Intel Corporation, Santa Clara, California, USA). Specimen images were gathered by randomly sampling 120 images from each of 10 herbaria (Desert Botanical Garden Herbarium [DES]; Marshall University [MUHW]; Morris Arboretum of University of Pennsylvania [MOAR]; Morehead State University Herbarium [MDKY]; Muhlenberg College [MCA]; Florida State University’s Robert K. Godfrey Herbarium [FSU]; University of South Carolina, A. C. Moore Herbarium [USCH]; University of Maryland, Norton‐Brown Herbarium [MARY]; Lynchburg College, Ramsey‐Freer Herbarium [LYN]; University of Tennessee, Chattanooga [UCHT]) accessed through the SouthEast Regional Network of Expertise and Collections (SERNEC) data portal (https://sernecportal.org; accessed 15 September 2020). The included herbaria were selected based on ability to provide high‐resolution images and the use of a uniform catalog number format. Image files were saved under collection code subdirectories and named according to the record’s universally unique identifier (UUID). Next, each image was inspected and those without a visible barcode were removed from the test data set. From the remaining images, 100 were randomly selected from each collection’s subdirectory and converted to grayscale.

Five alternative barcode decoding methods were evaluated alongside VARP for decoding success rate and execution time. Each method was tested against the entire test data set. Three of these methods used the Zbar library accessed through the unmodified PyZbar library. These methods included both strategies included in the Natural History Museum of London’s Gouda package (https://github.com/NaturalHistoryMuseum/gouda) (“Gouda‐resize” and “Gouda‐roi”) and a method used by the Tennessee Herbaria Consortium (“THC‐rotation”). Similar to VARP, the Gouda‐roi method takes a proactive preprocessing approach using OpenCV to identify regions of interest. The Gouda‐roi approach differs from VARP in that it attempts to identify the entire barcode region through a series of thresholding, horizontal blurring, and vertical blurring. Gouda‐resize is a reactive method that attempts to repeatedly upscale the image until line scanning is successful. The THC‐rotate method is also a reactive strategy, which attempts to overcome rotational variance by repeatedly rotating the full‐scale image until line scanning is successful. Two of the methods evaluated use the ZXing library through the Python wrapper python‐zxing (https://github.com/dlenski/python‐zxing), which was evaluated with and without the “try_harder” parameter set (“ZXing‐th” and “ZXing,” respectively). The ZXing method utilizes localized thresholding techniques that compare relative pixel intensity with neighboring pixels to convert the image to binary (i.e., black and white) before applying multiple format‐specific line‐scanning algorithms. The ZXing‐th method uses the same strategy as ZXing but with more computationally intensive thresholding and line scanning.

Procedural errors during digitization, such as synchronization errors, may cause images to be associated with incorrect catalog numbers. For example, three of the records in the test data set had mismatched barcode and catalog numbers. Because of this, successful decoding was determined by pattern matching using collection‐specific regular expressions. If a decoded value matched the format expected from its collection’s catalog numbers, then it was considered a success. To account for potential extraneous barcodes in images, only one correctly formatted result was necessary for an image to be considered successfully decoded. The results of these evaluations are reported in Table 1 as “Decoding success.” Among the 1000 images in the test data set, 21 were not successfully decoded by any method. The VARP method had the highest success rate, decoding 965 images, whereas the Gouda‐resize method successfully decoded 917 images. All of the other methods (i.e., THC‐rotation, ZXing‐th, Gouda‐roi, and ZXing) decoded less than 75% of the test data set. Due to a 0% success rate, the ZXing method without the “try_harder” parameter (i.e., ZXing) was omitted from execution time tests.

**TABLE 1:** The success rates and execution times of multiple methods decoding barcodes from 1000 herbarium specimen images.

Method	Decoding success (%)	Successful execution time^a^, sec	Failure execution time^b^, sec	Combined execution time^c^, sec
Gouda‐resize	917 (91.7)	1.689	27.427	1.934
Gouda‐roi	479 (47.9)	0.367	0.339	0.356
THC‐rotate	742 (74.2)	1.469	6.936	2.273
VARP	965 (96.5)	0.612	1.106	0.617
ZXing‐th	643 (64.3)	3.281	2.173	2.198
ZXing^d^	0 (0)	—	—	—

^a^ Successful execution time is the median time of all successes for the corresponding method.

^b^ Failure execution time is the median time of all failures for the corresponding method.

^c^ Combined execution time is the median time of all decoding attempts for the corresponding method.

^d^ The ZXing method without the “try hard” parameter (i.e., ZXing) was omitted from execution time tests because of the initial 0% decoding success rate.

To evaluate execution times, each tested method was used to decode every image five times. The order in which methods were evaluated was randomized for each image. The median time among all five attempts was then documented as that method’s execution time for that specific image. All methods were instantiated before testing to omit dependency loading from the recorded times. Because the VARP and THC‐rotation methods expect previously loaded image data while the remaining methods handle reading image files internally, helper functions were used to ensure that reading the images from disk was included in all time tests. The median time of each method’s successfully decoded images is reported in Table 1 as the “successful execution time.” The median time of each method’s failed decoding attempts is reported in Table 1 as “failure execution time.” Finally, the combined execution times for each method’s successful and failed decoding attempts are reported in Table 1 as the “combined execution time.” The Gouda‐roi method had the fastest combined execution time of 0.356 seconds, while VARP’s combined execution time was 0.617 seconds. The combined execution time for each other method (i.e., Gouda‐resize, ZXing‐th, and THC‐rotation) exceeded 1.9 seconds.

Because VARP’s primary strategy is to reduce resolution without discarding barcode data, an additional analysis was performed to determine the median rate of resolution reduction when images are composited during VARP’s preprocessing. To perform this analysis, VARP’s decoding function was modified to return the proportion of total pixels in the source image to the total pixels in the composite image. This modified function was then applied to all 1000 images in the test data set, with the resulting proportions being stored as an array. The observed median resolution reduction was 98.5%.

## CONCLUSIONS

Vector‐assisted region proposals improved the speed and reliability with which barcodes can be decoded from herbarium images. The added complexity of VARP’s preprocessing is evidently justified given the superior decoding success rate of 96.5% and performant execution time of 0.617 seconds. The one method that was faster than VARP was the other preprocessing‐focused method, Gouda‐roi. Although it was the fastest method, with a median execution time of 0.356 seconds, Gouda‐roi only succeeded in decoding 47.9% of the test data set. Regardless, these results suggest that preprocessing images with the goal of identifying regions of interest can produce faster outcomes in this domain.

Gouda‐resize was the second most successful method, achieving an appreciable decode success rate of 91.7%. It is interesting to note, however, that this was not uniformly observed across the other reactive methods. Because Gouda‐resize and THC‐rotate utilized the same line‐scanning library, the disparity in success rates (91.7% and 74.2%, respectively) is likely attributable to how they handle failed attempts, with Gouda‐resize upscaling images after each failure while THC‐rotate rotated images. These results suggest that rotational variance plays a minimal role in overall success rates. Unsurprisingly, both of these reactive strategies (i.e., Gouda‐resize and THC‐rotate) were significantly slower than their counterparts, exhibiting widely divergent execution times between successes and failures. The low success rates for both methods using the ZXing library (i.e., ZXing and ZXing‐th) calls into question the accuracy of the implementation as well as the performance of the Python compatibility layer. Further testing of the ZXing methods outside of the Python environment may show improvements.

Although developed, tested, and discussed from the perspective of herbarium digitization of vascular plants, it is possible the VARP method could similarly improve digitization workflows for other taxonomic groups. There may also be implications for VARP outside of natural history digitization, but there are two considerations worth noting before adapting VARP for other domains. The first consideration is the untested assumption that the benefits of VARP’s complex preprocessing are only realized when a barcode occupies a small portion of a high‐resolution image. The second consideration is that VARP’s processing disassociates positional relationships. Both decoding backends evaluated (i.e., ZBar and ZXing) can identify the position of barcodes within images, a feature some workflows may find useful. This positional information is currently being discarded when VARP constructs the composite image. It is feasible that VARP could be modified to support this feature by retaining vector coordinates relative to their position within the composite image, but as this has not been requested for natural history digitization, it has not been explored.

VARP was developed in conjunction with other herbarium image analysis optimizations (Ledesma et al., 2020) to support ongoing development of HerbASAP, an open‐source project seeking to automate real‐time image post‐processing for herbarium digitization (https://github.com/CapPow/HerbASAP). Additionally, a simple‐to‐use command line interface (CLI) has been developed as a proof‐of‐concept. The CLI, as well as details concerning the test data set and performance evaluations, are available on GitHub (https://github.com/CapPow/VARP_supplimental ). These materials have been provided under the open‐source MIT license and may therefore be freely inspected, used, or adapted for other works.

## AUTHOR CONTRIBUTIONS

**Caleb Powell: Conceptualization (equal); Investigation (equal); Methodology (equal); Software (equal); Validation (equal); Visualization (equal); Writing – original draft (equal); Writing – review & editing (equal). Joey Shaw: Writing – review & editing (equal).**

C.P. conceptualized and designed the work, performed analysis, produced figures and tables, and wrote the original draft. J.S. provided funding, feedback on content, and contributed to review and editing.

### Open research badges

This article has earned an Open Data and Open Materials badges for making publicly available the digitally‐shareable data necessary to reproduce the reported results. The data is available at https://forsbase.unil.ch.

## Data Availability

Additional supporting data, as well as a simplified VARP implementation, are available through a public GitHub repository (https://github.com/CapPow/VARP_supplimental).
